# Mitogenomic Alterations in Breast Cancer: Identification of Potential Biomarkers of Risk and Prognosis

**DOI:** 10.3390/ijms26178456

**Published:** 2025-08-30

**Authors:** Carlos Jhovani Pérez-Amado, Amellalli Bazan-Cordoba, Laura Gómez-Romero, Julian Ramírez-Bello, Verónica Bautista-Piña, Alberto Tenorio-Torres, Eva Ruvalcaba-Limón, Felipe Villegas-Carlos, Diana Karen Mendiola-Soto, Alfredo Hidalgo-Miranda, Silvia Jiménez-Morales

**Affiliations:** 1 Laboratorio de Innovación y Medicina de Precisión Núcleo “A”, Instituto Nacional de Medicina Genómica, Mexico City 14610, Mexico; 2 Programa de Maestría y Doctorado, Posgrado en Ciencias Bioquímicas, Universidad Nacional Autónoma de México, Mexico City 04510, Mexico; 3 Subdirección de Bioinformática, Instituto Nacional de Medicina Genómica, Mexico City 14610, Mexico; 4 School of Medicine and Health Sciences, Tecnológico de Monterrey, Mexico City 14380, Mexico; 5 Subdirección de Investigación Clínica, Instituto Nacional de Cardiología Ignacio Chávez, Mexico City 14080, Mexico; 6 Fundación de Cáncer de Mama, FUCAM, Mexico City 04980, Mexico; 7 Programa de Doctorado en Ciencias Biomédicas, Universidad Nacional Autónoma de México, Mexico City 04510, Mexico; 8 Laboratorio de Genómica del Cáncer, Instituto Nacional de Medicina Genómica, Mexico City 14610, Mexico

**Keywords:** breast cancer, case-control study, heteroplasmy, haplogroups, mitochondrial DNA, mtDNA mutations, mtDNA biomarkers

## Abstract

Alterations in the mitochondrial genome (mtDNA) have been shown to be key in cancer development and could be useful as biomarkers for diagnosis, prognosis, and treatment. To identify mtDNA variants associated with breast cancer, we analyzed the whole mtDNA sequence from paired tissues (tumor–peripheral blood) of women with this malignancy and from peripheral blood samples of healthy women. The mtDNA mutational landscape, heteroplasmy levels of the variants, and mitochondrial ancestry were established. Comparative analysis between cases and controls revealed significant differences in the number and location of variants, as well as in the heteroplasmy levels. Cases showed higher mutation number in *MT-ND5*, tRNAs, and rRNAs genes; increased proportion of missense variants; and elevated mtDNA content, than controls. Notably, a high blood mtDNA mutational burden (OR = 3.83, CI: 1.89–7.95, *p* = 5.3 × 10^−5^) and five mtDNA variants showed association with the risk of breast cancer. Furthermore, a low tumor mutational burden (HR = 7.82, CI: 1.0–63.6, *p* = 0.05) and the haplogroup L (HR = 12.16, CI: 2.0–72.8, *p* = 0.0062) were associated with decreased overall and disease-free survival, respectively. Our study adds evidence of the potential usefulness of mtDNA variants as risk and prognosis biomarkers for breast cancer.

## 1. Introduction

Breast cancer is the most common tumor in women around the world [1,2,3]. Nowadays, the unceasing campaigns to prevent and early detect this disease have contributed to improving the 5-year survival rates; however, in Mexico, as in many countries of the world, breast cancer is the leading cause of cancer-related deaths among women [3,4]. Breast cancer is a diverse group of diseases that is clinically and biologically heterogeneous and classified into four subtypes based on receptor protein expression and gene expression profiles. These subtypes are luminal A, luminal B, HER2-enriched, and triple-negative (also known as basal-like) [5,6]. Although the identification of the molecular subtypes of breast cancer is essential for an appropriate clinical and therapeutic approach, late diagnosis and differential response to treatment still represent the main limitations for increasing survival rates of these malignancies [3]. Furthermore, many of the biological mechanisms underlying the development and progression of breast cancer are still unknown [7].

Due to mitochondria’s involvement in numerous cellular processes, such as ATP synthesis via oxidative phosphorylation (OXPHOS), metabolic pathways, production of reactive oxygen species (ROS), apoptosis, and regulation of signaling pathways involved in cell growth and development, their study is highly relevant in numerous human diseases [8,9]. Mitochondria possess their own genome, whose alterations have been shown to promote bioenergetic changes needed for cellular transformation and oncogenesis [8,10,11,12]. The mitochondrial genome (mtDNA) is a circular 16.5 kb double-stranded DNA that contains a total of 37 genes (2 rRNA, 22 tRNA, and 13 protein subunits) encoding essential subunits of the OXPHOS system. mtDNA is maternally inherited, presents a high mutational rate, and can be found in a large number of copies per cell. Because of this last feature, a cell can be homoplasmic if all its mtDNA molecules have the same sequence or heteroplasmic if it contains a mixture of mtDNA molecules with different sequences [8,13,14].

Although the mutational landscape of mtDNA has been described in several types of tumors [8,15,16,17,18,19], the role of these mutations in the oncogenesis and cancer progression processes is unknown, and it seems to depend on the type of mutation, its functional consequences, and the proportion of mutated mtDNA within a cell (heteroplasmy level) [8,14,17]. However, studies have suggested that specific mtDNA variants, such as A10398G and haplogroups (a group of variants that are inherited together without any recombination and correlate with the geographic origin of populations) as D, are associated with breast cancer and have been proposed as risk factors for tumor development. In addition, the number of somatic variants in tumors has been linked with the overall survival of breast cancer patients [8,20,21,22]. Recent evidence even suggests that alterations in mtDNA copy number are involved in tumor biology and could be a potential tool to early detect women with breast cancer [23].

Thus, to identify potential biomarkers in breast cancer, we analyzed the whole mtDNA of paired samples (tumor–peripheral blood) from women with breast cancer and peripheral blood (PB) samples of women without cancer. Our genomic approach allowed us to determine the mutational landscape of mtDNA from peripheral samples, heteroplasmy levels of the variants, mtDNA copy number (mtCN), mitochondrial ancestry, and identify specific variants associated with breast cancer. Additionally, it allowed us to establish the correlation among mtDNA variants and clinical variables of the disease, such as clinical stage, tumor subtype, metastasis, and overall survival.

## 2. Results

### 2.1. Study Population

We retrieved the complete mtDNA sequences of 92 paired breast tumor-PB samples analyzed in our previous work [18]. As we described previously, the ages of this cohort ranged from 33 to 92 years old ($\bar{x}$ = 53.83 ± 11.5). In 90 cases, tumors were classified with immunohistochemical (IHC) markers [3], being 63.3% (*n* = 57) luminal A, 24.4% (*n* = 22) luminal B, 5.6% (*n* = 5) HER2, and 6.7% (*n* = 6) triple negatives (Table 1). Additionally, we sequenced the mtDNA from 75 PB samples from healthy women with ages ranging from 37 to 66 years old ($\bar{x}$ = 48.1 ± 4.3). Regarding age, no significant differences between patients and controls were observed (*p* = 0.06).

### 2.2. Landscape of Mitochondrial DNA Germline Variants in Breast Tumors

The landscape of the somatic mutations in breast tumors was previously published by our group [18]; thus, in the present work, we focus on the study of the germline mtDNA variants in breast tumors. A total of 869 variable positions and 42.7 ± 11.4 mtDNA germline variants on average per tumor (range: 24–90 variants) were identified. Based on the variant type, 95.6% (*n* = 831) were single-nucleotide variants (SNVs), 2.2% (*n* = 19) deletions, and 2.2% (*n* = 19) insertions (Supplementary Table S1). According to gene location, 59.95% (*n* = 521) were located in protein-coding genes and 40.05% (*n* = 348) in non-coding regions. The D-Loop region (*n* = 167), *MT-ND5* (*n* = 89), *MT-ND4* (*n* = 62), *MT-CYB* (*n* = 56), and *MT-CO1* (*n* = 61) genes exhibited the highest number of variants. Missenses were the most frequent coding variants (48.6%, *n* = 253), followed by synonymous (46.0%, *n* = 240), stop gained (4.2%, *n* = 22), frameshift (0.8%, *n* = 4), inframe (0.2%, *n* = 1), and start lost (0.2%, *n* = 1) variants. Otherwise, 49.7% (*n* = 173) of the non-coding variants were located in rRNA and tRNA genes, 47.4% (*n* = 165) had regulatory functions, and 2.9% (*n* = 10) were intergenic (Supplementary Figure S1).

Heteroplasmy levels of the identified mtDNA germline variants were widely heterogeneous among tumors (from 0.8 to 95.0%), showing a mutant allele fraction (MAF) mean of 29.28%. Additionally, tumors had a mtDNA content mean of 38.96 ± 28.77 copies, while 30.36 ± 24.04 copies were detected in PB; although no significant differences among paired tumor–PB samples were observed (*p* = 0.086) (Supplementary Table S2 and Figure S2).

### 2.3. High Mutational Rate of Mitochondrial Genome from Peripheral Blood of Patients with Breast Cancer

Our analysis revealed that all patients with breast cancer carried mtDNA alterations in PB, and a mtDNA mutation rate of 52.3 muts/kb was estimated. mtDNA germline variants ranged from 27 to 109 ($\bar{x}$ = 46.1 ± 16.8) per patient. A total of 866 different mtDNA germline variants distributed throughout the entire genome was found. Overall, of the total variant types, 95.9% (*n* = 831) corresponded to SNVs, 2.2% (*n* = 19) were deletions, and 1.9% (*n* = 16) were insertions (Table 2). The highest number of variants was observed in the D-Loop region (*n* = 164), followed by the coding genes *MT-ND5* (*n* = 94), *MT-ND4* (*n* = 66), *MT-CYB* (*n* = 58), and *MT-CO1* (*n* = 54). Despite the fact that the highest mutational rate occurred in the D-Loop region (146.4 muts/kb), genes that transcribe tRNA, such as *MT-TT* (107.7 muts/kb), *MT-TC* (92.3 muts/kb), and *MT-TR* (62.5 muts/kb), and coding genes, such as *MT-ATP6* (72.1 muts/kb) and *MT-ATP8* (68.0 muts/kb), also presented high mutation rates.

**Figure 1:**
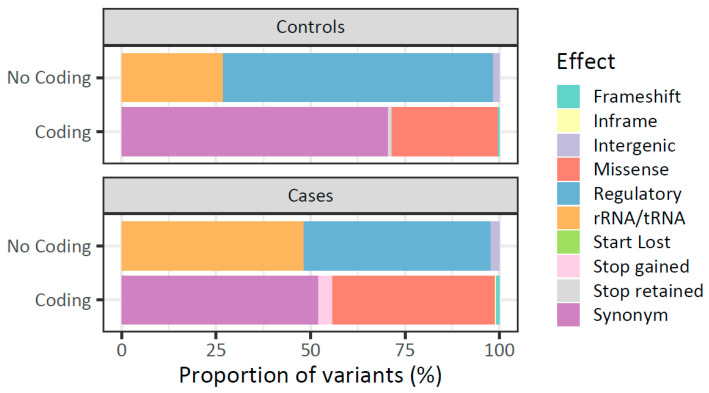
Functional effect of mtDNA variants in patients with breast cancer and healthy women. Distribution of mtDNA variants in peripheral blood of healthy women (Controls) and women with breast cancer (Cases) is represented. Variants are stratified by their location (coding and non-coding region) and in silico potential functional effect.

Based on the mtDNA germiline variants effect prediction, 62% (*n* = 534) were located in coding regions, of which 52.13% (*n* = 281) were synonymous, 43.23% (*n* = 233) were missense, 3.53% (*n* = 19) were stop-gained, 0.93% (*n* = 5) were frameshift, and 0.19% (*n* = 1) were inframe. The variants in non-coding regions represented 38% (*n* = 332) of the total variants, and those with regulatory effect were the most predominant (49.40%, *n* = 164), followed by those affecting tRNA and rRNA transcripts (48.19%, *n* = 160), and intergenic variants (2.41%, *n* = 8) (Figure 1).

### 2.4. Low Mutational Rate of Mitochondrial Genome from Peripheral Blood of Healthy Women

The mtDNA mutational rate in blood samples of controls was 24.2 muts/kb, and the mtDNA germline variants were in the range from 21 to 49 ($\bar{x}$ = 33.6 ± 6) variants per individual (Table 2). The identified variants were located in 401 positions distributed throughout the entire mtDNA. D-Loop region presented the greatest number of variants and the highest mutation rate (*n* = 119 variants, 106.3 muts/kb, respectively), which were similar to patients’ findings. In spite of the fact that coding genes such as *MT-CO1* (*n* = 32), *MT-CYB* (*n* = 28), and *MT-ATP6* (*n* = 26) presented a high number of variants, the *MT-TT* (76.9 muts/kb), *MT-ATP8* (38.8 muts/kb), *MT-ATP6* (38.2 muts/kb), and *MT-TL2* (28.6 muts/kb) genes showed the highest mutation rate.

Regarding the mtDNA location of the identified variants in healthy women, 59% (*n* = 238) and 41% (*n* = 167) were located in coding and non-coding regions, respectively. Synonymous coding variants showed the highest proportion (70.59%, *n* = 168), followed by missense (28.15%, *n* = 67) and frameshift (0.42%, *n* = 1) variant types. Of the total number of non-coding variants, 71.26% (*n* = 119) were regulatory, 26.95% (*n* = 45) occurred in rRNA/tRNA transcripts, and 1.80% (*n* = 3) were in intergenic regions (Figure 1).

### 2.5. The Landscape of mtDNA Germline Variants Differs Between Patients with Breast Cancer and Healthy Women

Our comparative analysis of mtDNA germline variants between patients with breast cancer and healthy women showed differences in the number and type of variants. The mtDNA mutation rate and the number of variants per individual (52.3 vs. 24.2 muts/kb, and $\bar{x}$ = 46.1 ± 16 vs. 33.5 ± 6, respectively) were higher in cases than controls (*p* = 6.2 × 10^−10^) (Table 2).

In terms of the types of variants (SNVs and indels), the differences observed between healthy women and patients were not statistically significant (*p* > 0.05). However, the proportion of missense variants and variants affecting rRNA/tRNAs was significantly higher in cases than controls (missense: 43.2% vs. 28.1%, *p* = 9.6 × 10^−5^; rRNA/tRNA: 48.19% vs. 26.9%, *p* = 0.0014). Otherwise, the proportion of synonymous and regulatory variants was lower in patients than healthy women (synonymous: 52.13% vs. 70.59, *p* = 0.0013; regulatory: 49.40% vs. 71.26%, *p* = 2.72 × 10^−5^). Stop-gained and in-frame variants were only detected in patients.

Although variants were distributed throughout the entire mtDNA, the genes *MT-ND5* (*p* = 0.011), tRNA (*p* = 0.022), and rRNA (*p* = 0.045) were significantly more mutated in patients with breast cancer in comparison to healthy women. Otherwise, the D-Loop region was significantly less mutated in cases than controls (*p* = 2.7 × 10^−5^), and *MT-TF*, *MT-TP*, and *MT-TS1* tRNA genes were exclusively mutated in controls (Table 3).

### 2.6. Higher Heteroplasmy Variation in Peripheral Blood of Patients with Breast Cancer than Healthy Women

The MAF estimation allowed us to determine the heteroplasmy levels of all mtDNA germline variants. In blood samples of patients, heteroplasmy levels ranged from 0.015 to 94.8% ($\bar{x}$ = 10.8%). Of the total variants, 66.5% (*n* = 576) were heteroplasmic (MAF ≤ 95%) and 33.5% (*n* = 290) homoplasmic (MAF > 95%). The heteroplasmic variants showed different heteroplasmy levels: high heteroplasmy (MAF = 20–95%, *n* = 54), low heteroplasmy (MAF = 0.5–20%, *n* = 222), and very low heteroplasmy or rare variants (MAF < 0.5%, *n* = 370).

In relation to the mtDNA germline variants of the control group, we observed a lower range of heteroplasmy (from 22.2 to 95.0%, $\bar{x}$ = 63.3%) than in cases. A total of 78.8% (*n* = 316) of these variants were homoplasmic and 21.2% (*n* = 85) were heteroplasmic. Notably, we did not identify variants with low heteroplasmy degree or rare variants in this group. The distribution of heteroplasmic and homoplasmic mtDNA germline variants was statistically different (*p* < 2.2 × 10^−16^) between cases and controls (Table 4).

Additionally, we observed that 30.77% (*n* = 212) of mtDNA germline variants presented differences in the heteroplasmic levels among individuals; even so, some of the carriers were homoplasmic for these variants, which were differentially distributed between cases and controls (165 vs. 47 variants, respectively; *p* = 0.0015).

We identified 208 mtDNA germline variants shared between cases and controls, which showed a wide range of heteroplasmy levels among individuals. Overall, shared variants, 85.6% (*n* = 178), were detected in more than two subjects. Of these, we observed that 29.2% (*n* = 52) showed lower heteroplasmy levels in cases compared with controls; in 4.5% (*n* = 8), heteroplasmy levels were higher in cases than in controls, and 66.3% (*n* = 118) did not present changes in heteroplasmy levels in both groups.

**Figure 2:**
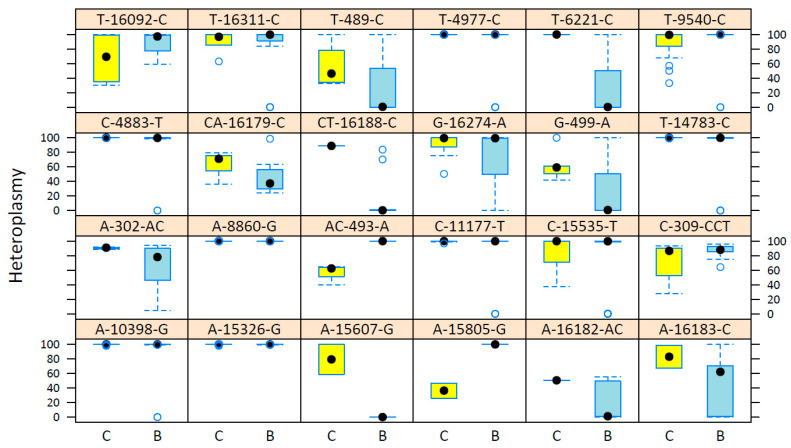
Heteroplasmy level distributions of mtDNA germline variants in peripheral blood of patients with breast cancer and healthy women. Each box represents a variant. The heteroplasmy levels are reported on the y-axis, and the evaluated groups are shown on the x-axis (C = control, B = breast cancer). Points represent individuals and bars represent heteroplasmy levels (yellow: control, blue: case). Variants detected in at least 10 individuals are displayed.

Interestingly, 35 variants showed significant differences between cases and controls in their heteroplasmy levels. Most of the variants (*n* = 31) were heteroplasmic in cases, but homoplasmic in controls. For example, the heteroplasmy of A16183C was higher than 67.4% in controls, while, in most cases, it was lower than 70%. On the other hand, the AC493A and A15805G variants were homoplasmic (>95%) in cases, but heteroplasmic (30–70%) in controls (Figure 2).

### 2.7. Association Analysis Between Mitochondrial DNA Variants and Breast Cancer

#### 2.7.1. The Mitochondrial Mutational Burden in Peripheral Blood Increases Risk for Breast Cancer

**Figure 3:**
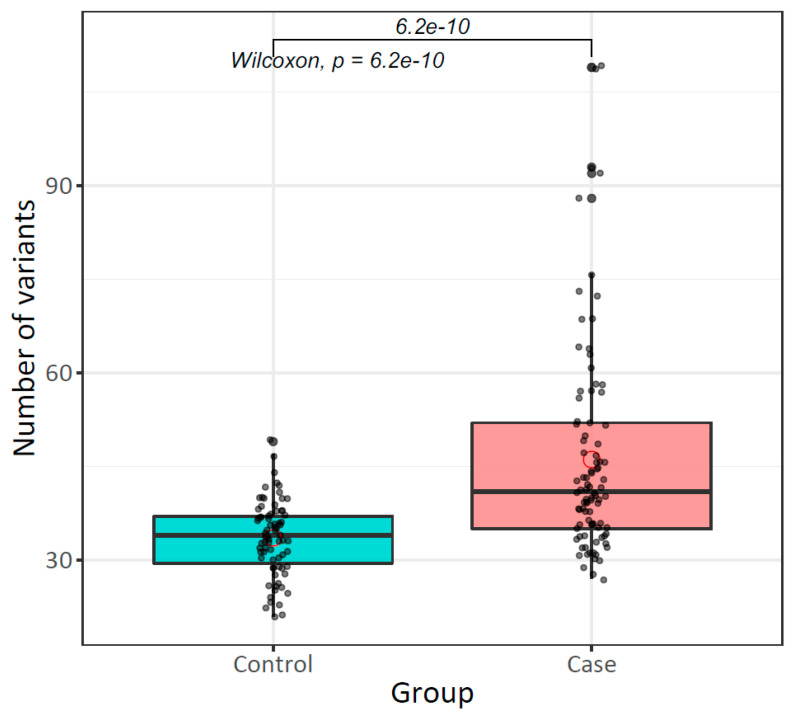
Mitochondrial DNA mutational burden in peripheral blood of patients with breast cancer and healthy women. The distribution of the total number of mtDNA germline variants identified in the peripheral blood of both groups is represented. Each black dot corresponds to the total number of variants per individual, while red circles represent the mean of total variants per group.

Based on the higher number of mtDNA germline variants per individual observed in cases than in controls (Figure 3), we investigated whether mitochondrial mutational burden was a risk factor for breast cancer. The median value of the number of mtDNA variants identified in controls (34 variants) was used as a cut-off point to categorize individuals with low (<34 variants) or high (≥34 variants) mutational burden. We observed that a high mitochondrial mutational burden in PB increases the risk of breast cancer (OR = 3.83, CI: 1.89–7.95, *p* = 5.3 × 10^−5^) (Table 5).

#### 2.7.2. Mitochondrial DNA Germline Variants Are Associated with Breast Cancer Development Risk

Comparing cases and controls, we identified 208 shared mtDNA germline variants between both groups, and variants that were unique for patients (*n* = 658) or controls (*n* = 193). Regarding shared variants, only A1438G and A15326G (located in *MT-12S* and *MT-CYB*, respectively) were present in all analyzed subjects. Relating to exclusive variants of patients, 31.2% (*n* = 205) occurred in more than two individuals, and the most frequent showed proportions between 6.5 and 11.9%. In contrast, variants detected solely in the control group showed high heterogeneity among women, and only 9.7% of them were carried by more than one individual, with the C309CCCT, located in the D-Loop region, being the most frequent (5.3%) variant. Another interesting finding was that the average levels of heteroplasmy of the shared variants between the cases and controls were very similar to those observed in the unique variants detected in healthy women (89.36% and 93.35%, respectively), contrasting with the low levels of heteroplasmy observed for the unique variants in cases (44.14%).

Based on the knowledge that mtDNA variants have been associated with the risk of breast cancer, we performed a case-control association analysis including all shared mtDNA germline variants. We identified 25 (12%) shared variants, for which the frequencies between both groups were significantly different. The A16183C (OR = 10.7, CI: 2.5–97.3, *p*-adj = 2.3 × 10^−4^), C14766T (OR = 18.8, CI: 2.7–816.3, *p*-adj = 3.5 × 10^−3^), and C7028T (OR = 17.1, CI: 2.4–746.2, *p*-adj = 0.02) mtDNA germline variants increased the risk of developing breast cancer. In contrast, A263G (OR = 0.2, CI: 0.1–0.4, *p*-adj = 1.5 × 10^−4^) and A235G (OR = 0.32, CI: 0.2–0.6, *p*-adj = 0.02) were identified as protective variants to this neoplasia (Table 6).

The mtDNA variants associated with breast cancer were located mostly in the D-Loop (*n* = 3 variants) region and the *MT-CO1* (*n* = 1 variant) and *MT-CYB* genes (*n* = 1 variant). According to the functional effect, the C14766T and C7028T coding variants were missense and synonymous, respectively. Regarding the A263G, A235G, and A16183C non-coding variants, only A263G and A235G showed a potential regulatory function because they were located in the H-strand replication origin region (OHR), binding sites for mitochondrial transcription factor (mtTF1), and conserved sequence blocks (CSBs).

Another relevant observation is that the heteroplasmy levels of the mtDNA variants associated with breast cancer differed between the studied groups. For instance, the A16183C variant, located in the D-Loop region, showed MAF average values of 50.6% (from 0.1 to 99.8%) and 83.1% (from 67.4 to 98.7%) in the cases and controls, respectively.

#### 2.7.3. Higher Peripheral Blood Mitochondrial DNA Content in Patients with Breast Cancer than Healthy Women

The mtCN in peripheral blood samples from patients and controls showed statistically significant differences (26.2 ± 17.6 vs. 18.5 ± 11.5 copies, respectively; *p* = 0.021), but no association between mtDNA content and the risk for breast cancer developing (OR = 1.9, CI: 0.96–3.78, *p* = 0.066) was observed (Supplementary Tables S3 and S4, Figure S3).

### 2.8. Native American Mitochondrial Haplogroups Were Enriched in Patients with Breast Cancer and Healthy Women

In terms of mitochondrial ancestry, Native American haplogroups (A: 58.7%, C: 14.7%, B: 12%, and D: 8%) were the most frequent in control group, while European (U: 2.7%, R: 1.3%, and T: 1.3%) and African (L: 1.3%) haplogroups occurred with less frequency.

In patients, the most common haplogroups were also Native American origin (91.3%, n = 84). Nevertheless, in contrast to the control group, the African haplogroup was slightly more frequent than the European haplogroups (5.4% vs. 3.3%, respectively). The frequencies of the identified haplogroups were 44.6%, 22.8%, 12%, 12%, 5.4%, 2.2%, and 1.1% (A, B, C, D, L, H, and J, respectively).

By comparing cases and controls, we observed a higher frequency of B (22% vs. 12%), D (12% vs. 8%), and L (5.4% vs. 1.3%) haplogroups in patients than in controls. Moreover, T and R haplogroups were identified exclusively in controls, while H and J haplogroups were exclusive in patients. However, differences were not statistically significant (*p* > 0.05), and no association with the risk of breast cancer was observed (Supplementary Table S5).

### 2.9. Tumor Mitochondrial DNA Mutational Burden Is Associated with Overall Survival in Breast Cancer

**Figure 4:**
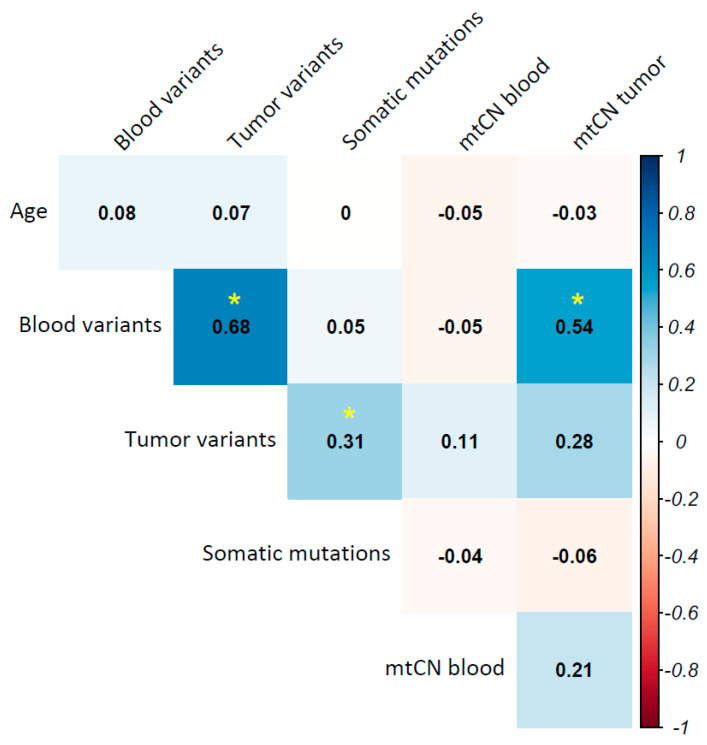
Correlation among mitochondrial DNA alterations in breast cancer. The correlation of mitochondrial alterations analysis (variants in blood, variants in tumor, somatic mutations, mtCN in blood, and mtCN in tumor) in patients with breast cancer is shown. The right gradient color bar represents the degree of correlation (Spearman Correlation) among each alteration. Blue: positive correlation, red: negative correlation, yellow asterisks (*) indicate statistical significance (*p* < 0.05).

A correlation analysis between mtDNA alterations and clinical variables of breast cancer, such as molecular subtype, clinical stage, metastasis, and death, was performed. mtDNA alterations included blood and tumor mutational burden (total variants per individual), total of somatic mutations, and mtCN in blood and tumor. The age of patients was accounted for based on their reports showing an association between mitochondrial mutation rate and aging; however, no correlation was observed (*p* > 0.05). Blood mutational burden correlated with both tumor mutational burden (R = 0.68, *p* = 6.5 × 10^−13^) and tumor mtCN (R = 0.54, *p* = 0.022). Additionally, a correlation between tumor mutational burden and the total of somatic mutations was observed (R = 0.31, *p* = 0.0003) (Figure 4).

**Figure 5:**
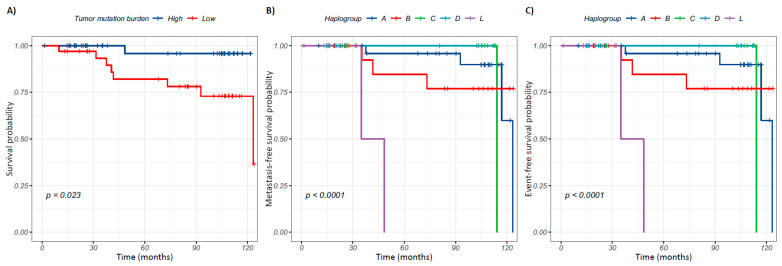
Association of tumor mtDNA mutational burden and mitochondrial haplogroups with breast cancer prognosis. (**A**) Overall survival analysis stratified by mitochondrial mutational burden in tumor (low: <39 variants and high: ≥39 variants). (**B**) Metastasis-free survival analysis stratified by haplogroups. (**C**) Event-free survival analysis stratified by haplogroups. Kaplan–Meier curves: vertical lines (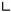) indicate the event (death, metastasis, death and/or metastasis, as appropriate), while crosses (+) indicate patients with censored follow-up.

The association of mtDNA alterations with clinical variables of breast cancer was evaluated (Supplementary Table S6). A significant reduction in mutational burden in blood (*p* = 0.04) and tumor (*p* = 0.0025) was observed in patients who died. To determine if tumor mutational burden is associated with the prognosis of patients, an over-survival analysis was carried out. For this, patients with an average clinical follow-up of 64 months (from 1 to 124 months) were included, of which 11.84% (*n* = 9) died, 14.47% (*n* = 11) developed metastasis, and 17.11% (*n* = 13) presented one or both events. Patients were categorized with a somatic, tumoral, or blood mutational burden that was high or low using the median value of variants per individual as a cut-off point (Supplementary Table S7). Kaplan–Meier analysis showed that patients with less than 39 mtDNA variants in the tumor had a poor prognosis (*p* = 0.023) (Figure 5A). Mantel–Cox regression analysis estimated that a low tumor mutational burden increases the risk of death (HR = 7.82, CI: 1.0–63.6, *p* = 0.05).

### 2.10. Mitochondrial Haplogroups Are Associated with Prognosis in Breast Cancer

The association between mitochondrial haplogroups and clinical variables of breast cancer was also estimated. Our analysis did not show an association among mitochondrial haplogroups nor with breast cancer molecular subtype, tumor grade, or clinical stage (*p* > 0.05). However, an association between mitochondrial ancestry and the prognosis of the patients was observed. Survival analysis included the most frequent haplogroups identified (A, B, C, D, and L). Patients with breast cancer carrying the L haplogroup had a poor prognosis in comparison with patients bearing the D haplogroup (*p* = 6 × 10^−6^). Mantel–Cox regression estimated that the L haplogroup increases the risk for metastasis development 27.4-fold in patients with breast tumors (HR = 27.35, CI: 4.5–166.1, *p* = 0.0003). Finally, an association between mitochondrial ancestry and event-free survival was also observed, with patients carrying the L haplogroup being at a higher risk (12.16-fold) of death or developing metastasis (HR = 12.16, IC: 2.0–72.8, *p =* 0.0062) (Figure 5).

## 3. Discussion

Breast cancer is currently a serious worldwide public health problem, with incidence and mortality rates constantly increasing [4,7]. Although there is a vast amount of research around this neoplasia, the current breast cancer screening tests and early detection tools are still limited for detecting the disease or its precursor lesion at an early stage prior to the onset of symptoms. Thus, several studies have focused on the identification of biomarkers that can be used for early diagnosis of breast cancer, to predict its progression, or as a therapeutic target. Most of these investigations have been carried out through analyzing nuclear DNA; however, studies of the mitochondrial genome have shown enormous potential for this purpose. The mtDNA is essential for adequate maintenance of cellular bioenergetics, and it is well recognized that alterations in its sequence and content have been associated with the metabolic reprogramming of tumor cells, promoting their survival and progression [8,14]. However, the clinical implications of these variations are uncertain in most types of cancer [16,17,22,24,25].

The aim of this study was to determine the landscape of mitochondrial alterations in PB of patients with breast cancer and to identify biomarkers with potential clinical applications. For this, we analyzed the complete mtDNA of paired (blood–tumor) samples of breast cancer patients and blood samples of healthy women. Most of the studies around mitochondrial oncogenomics are focused on the identification of somatic mutations in tumors through their comparison with a reference tissue, as normal adjacent tissue or/and PB [11,19,26,27,28,29]. Since we have already reported the landscape of mutation signatures of breast tumors [18], we have focused on the germline variants.

The similar mutational rate of breast cancer tumors and paired PBs (52.4 vs. 52.3 muts/kb, respectively) observed in our analysis contrasts with previous studies, in which a differential mutational rate between tumor and normal tissue, and a higher frequency of mutations in *MT-ATP6*, *MT-ND3*, and *MT-CYB* genes have been reported [15,16,19,22,28,29,30,31]. Further, a wide range of heteroplasmy levels of the mtDNA germline variants across tumors and a higher tumoral mtDNA content than their matched PB samples was detected. These findings can be explained by the hypoxic and oxidative environment in which the tumors are immersed [9,14,18,24]. High concentrations of ROS damage mtDNA and promote dysfunction of essential OXPHOS system protein subunits, changing the cellular bioenergetic and activating signaling pathways implicated in the adaptation and progression of tumors [13,22].

Our case-control approach allowed us to identify noticeable differences between the two groups. We found a higher mtDNA mutation rate, wider ranges in heteroplasmy levels, and elevated mtDNA content in PB from patients than controls. As well, significant differences in the mutation type distribution and the location of the mtDNA variants between the two groups were observed. For instance, mtDNA variants in the *MT-ND5*, tRNA, and rRNA genes, plus non-synonymous type variants, were enriched in patients, but variants in the D-Loop and regulatory type were more frequent in the controls than in their counterparts. In contrast with these findings, a study performed in patients with breast cancer and healthy women from Sri Lanka did not report significant differences between the cases and controls, which could be due to the small sample size or ancestry background [32]. However, in accordance with our study, these authors also found a higher proportion of missense variants (58.3%) in patients with breast cancer than in controls [32]. No data are available to explain the biological meaning of our findings. Nonetheless, it has been reported in lung carcinomas that the *MT-ND5* gene is also highly mutated. Functional experiments using base editing tools to generate mtDNA truncating mutations in the *MT-ND5* gene showed decreased expression of complex 1 accessory proteins encoded in the nDNA [33,34]. Additionally, depending on the heteroplasmy levels, mutations can decrease proton-motive potential, modify the NAD+/NADH ratio, and alter concentrations of malate, lactate, fumarate, and arginosuccinate, promoting a metabolic imbalance that could impact the antitumor response [34]. Mutational enrichment in mitochondrial rRNA and tRNA genes of patients with breast cancer suggests a partial impairment in mitochondrial protein synthesis derived from a carcinogenic environment. Studies indicate that these genes retain and better tolerate deleterious variants in comparison to the mtDNA coding region, and even loss of function could be compensated through negative selection of the variants [35,36,37]. Mutations in mitochondrial tRNAs could alter the secondary structure and stability of transcripts, affecting tRNA–amino acid coupling and its insertion into the ribosome. Different types of cancer have shown an enrichment in mitochondrial variants in tRNAs, which are clustered specifically at the anticodon region [15]. Specifically, in patients with breast cancer, mutations in tRNAs such as G1606A (tRNAVal), A4300G (tRNAIle), T7505C (tRNASer1), A14693G (tRNAGlu), and G15927A (tRNAThr) have shown decreased mtDNA content and ATP production, suggesting that these regions are critical for oncogenesis [38].

Furthermore, heteroplasmy is a complex phenomenon that determines pathological phenotypes and their severity levels [16,24,25,36,39]. We observed that mtDNA from the PB of cases with breast cancer showed a wider range of heteroplasmy and a lower proportion of homoplasmic variants than in the controls. In addition to this, the detection of variants with heterogeneous heteroplasmy levels between individuals, such as T16092C, T489C, and A16183C, suggests the presence of mutational events regulated by selection mechanisms based on the functional effect of the mutation and its repercussion in tumoral bioenergetics [27,28]. mtDNA analyses in lung, colon, and liver carcinomas have found that heteroplasmy patterns are tissue- and region-specific, and arise from selection pressures caused by the biochemical and environmental context [24,36]. For example, coding and RNA transcription variants have been identified at low levels of heteroplasmy, and the *MT-ND5* gene has proven to be the most susceptible to heteroplasmic changes [11,33,34,40,41,42]. Specific heteroplasmy patterns have been observed for each cancer type [16,24]. The effect and functional mechanisms of mtDNA variants and their heteroplasmy levels in cancer, including breast cancer, have not been sufficiently explored, mainly due to technical limitations in the selection of specific genotypes. Research on tumors has proposed that the deleterious effect of the non-synonymous mtDNA variants can be compensated with modifications in heteroplasmy levels, promoting mitochondrial dynamics that are reflected as changes in mtDNA copy number or even through the co-gain of other mutations [9,11,16,17,23,24,36,41]. However, the explanation for the behavior of the mtDNA variants in PB of patients with breast cancer must be further investigated.

What has been interesting is that case-control studies analyzing PB mtDNA alterations in breast cancer and other types of neoplasia have shown differences between patients and healthy subjects, even years before cancer diagnosis [29,43,44,45]. For instance, Hofmann et al., by studying the mtDNA copy number in renal cell carcinoma pre-diagnostic leukocytes, found that a high mtDNA content is associated with an increased risk for this neoplasia even six years prior to diagnosis [44].

Our analyses suggest that mtDNA mutational burden and the presence of specific variants in PB could have an impact on the identification of women at risk of developing breast cancer, as well as to know the prognosis of the patients. At present, there is no evidence regarding mtDNA mutational burden in PB and the increased risk of breast cancer; however, a higher proportion of mtDNA variants in cases with respect to controls has been previously reported [27,29,46]. A detailed comparison of mtDNA variation between women with breast cancer and controls allowed us to identify potential variants associated with the risk of developing breast tumors. Although studies with similar approaches have been performed, the association of these variants with the risk of this malignancy has not been reported [32,43].

Around 64% of the mtDNA germline variants with differential frequencies between patients with breast cancer and healthy women detected in the present work have been found in other types of cancer, such as oral, nasopharyngeal, ovarian, endometrial, prostate, lung, colorectal, melanoma, glioblastoma, thyroid, gastric, and pancreatic [47]. The association between mtDNA variants and breast cancer risk is debatable, and it has been suggested that these findings could be influenced by demography, as well as clinicopathological and genetic variables, such as tumor origin, molecular subtype, ethnic group, and the co-occurrence with other mtDNA variants [20,48,49,50,51,52,53].

Based on our data and other works, the detection of mtDNA alterations in PB seems to be a promising tool to evaluate the risk of breast cancer developing. A recent study in Swedish women with breast cancer identified concordance among mutations in the mtDNA of paired samples (tumor–PB) prior to diagnosis, suggesting that these genomic changes can be detected before tumor development [29]. Furthermore, our findings, indicating that tumor mtDNA mutational burden is associated with overall survival, suggest that the use of mtDNA variants as prognosis biomarkers could be expanded even to women who have already been diagnosed with breast cancer, allowing for the identification of those patients with poor prognosis. The association of mtDNA mutational burden and cancer prognosis has been previously reported in patients with breast and colorectal tumors [15,22], although these studies have only considered somatic mutations. Other authors have found that specific mutated regions (e.g., D-Loop) and mutational profiles are associated with poor overall survival, disease-free progression, and recurrence-free survival in lung and liver cancer [26,33]. The implementation of mitochondrial mutational burden as a potential tool to determine the prognosis of breast cancer would allow opportune therapeutic intervention in patients; however, analyses in large cohorts that validate these findings are required.

Regarding haplogroups, there is increasing evidence showing an association among some of them with cell biochemistry changes [54]. Studies suggest that haplogroups could modify the incidence, cell mass, invasion capacity, and tissue heterogeneity of tumors through the activation of signaling pathways that alter the antitumor immune response, thus promoting a favorable environment for cancer cells’ progression [15,55,56,57,58,59]. For instance, it has been reported that haplogroups J and T increase the risk of developing gliomas [54], while haplogroup C has been associated with the development of gastric cancer [60].

Our results showed a higher frequency of haplogroups B and D in patients with breast cancer in comparison with controls, but no statistical differences were observed. Haplogroup B has been reported to increase 2-fold the risk of developing cervical cancer in Mexican women [61], and haplogroup D has been reported as a risk factor for breast cancer in Asian and European populations [8].

Additionally, we observed that haplogroup L increases the risk of presenting a metastatic event and/or death. Paradoxically, functional studies have found that haplogroup L increases the expression of genes encoding respiratory complexes and correlates with a lower rate of ATP turnover and production of ROS that give rise to a more efficient OXPHOS system [62]. However, the preservation of a functional OXPHOS system could support optimal cellular bioenergetics for tumor growth and dissemination. Furthermore, we cannot discard that the additive effect of other variants acquired during carcinogenesis also contributes to the unfavorable prognosis of the patients. Due to the sample size, our findings have low statistical power; thus, additional studies increasing the number of haplogroup L carriers are needed to know whether haplogroup L is associated with the risk and progression of breast cancer and if it has clinical relevance. According to other authors, we suggest that mitochondrial haplotypes could be the effector and modifier of tumorigenesis and its progression, through mediating metabolism, redox state, cell death, and immune response [15,58,59].

Because of the intrinsic characteristics of mtDNA, mitochondrial oncogenomics is an extremely complex field of research, displaying huge technical and analytical challenges. For example, the incorporation of nuclear mitochondrial segments (NUMTs) could alter the determination of heteroplasmy levels [63,64,65]; thus, we enriched the mtDNA by a long PCR before sequencing it. Also, sequencing was performed at a depth coverage greater than 300x per base, which, in addition to the use of Strelka2 algorithms for variant calling, allowed us to detect mutations with a minimum proportion of 0.02%. In fact, due to its ability to detect somatic mutations in low proportions, Strelka2 has been proposed as a valuable tool for cancer mutational clonality studies [66]. However, heteroplasmy is a complex phenomenon where numerous biological and technical variables intervene [65,67]. The integration of single-cell sequencing strategies will allow more accurate analysis of heteroplasmy [63,64,68,69] and conclude the correlation between heteroplasmy, breast tumor development, and clinical variables.

For clarifying the impact of mitochondrial alterations in cancer, it is required to design experimental and multivariate studies that integrate omics and functional approaches [15,70,71]. High-throughput technologies that allow for the study of genomics, transcriptomics, proteomics, metabolomics, epigenomics, etc., in a nuclear context, have made great progress and could be applicable in the mitochondrial context [24,41,72,73,74].

Although experimental approaches have to be developed for deciphering the biological implications of haplogroups and specific mtDNA variants in the oncogenesis processes, it is highly valuable that a wide variety of studies have indicated that mtDNA variants could be used as biomarkers of risk, diagnosis, and prognosis in cancer, including breast cancer [29,40,41,42,75,76].

The evaluation of these mitogenomic biomarkers in a minimally invasive and easily accessible sample, such as PB, increases their potential clinical implementation. Why these mtDNA alterations can be detected in the peripheral blood of patients with breast cancer remains a cornerstone question. Several studies have suggested that an mtDNA alteration detected in peripheral blood may come from tumor cell-free mtDNA, tumor extracellular vesicles, mitochondria derived from circulating tumor cells, or due to alterations produced by a highly oxidative microenvironment, which could be characteristic of patients with breast cancer [75,77,78,79]. In lung carcinoma, mtDNA variants identified in tumors are also detected in circulating tumor DNA from the plasma in 90% of patients, even with concordant heteroplasmy levels [42]. Estimating the mitochondrial mutation rate in plasmatic extracellular vesicles from patients with pancreatic cancer showed an increased number of variants in *MT-ND1*, *MT-CYB,* and *MT-ND5* genes in comparison with controls [75]. The presence of alterations in mtDNA that we observed in the PB of patients could also be explained by hormonal stimulation associated with breast tumors. Studies in endometrial carcinoma have shown that the presence of estrogens and their metabolites promotes the production of ROS, which increases mtDNA mutations and probably contributes to cancer origin and progression [80].

In summary, our findings suggest that certain mitochondrial DNA variants may be potential biomarkers of risk and prognosis for breast cancer; however, these data require additional validation. Longitudinal studies that include an independent cohort that comprises a larger number of cases and controls, which should be collected with a rigorous clinical and demographic characterization, will allow us to decipher the mitochondrial mutational dynamics and their contribution to breast tumorigenesis, as well as to determine their value as a risk predictor of this malignancy [64]. If the above is achieved, strategies for the opportune detection of breast cancer could be implemented, and those women already diagnosed could have more effective therapeutic options.

## 4. Materials and Methods

### 4.1. Study Population

mtDNA sequences of paired tumor–peripheral blood (PB) samples from 92 women diagnosed with breast cancer attended at the Instituto de Enfermedades de la Mama FUCAM (Mexico City, Mexico) were included. Patients with antineoplastic treatment prior to mastectomy were excluded. Breast tumors were classified using IHC markers as luminal A (LA), luminal B (LB), HER2-positive (HER2), and triple negative (TN). Additionally, 75 peripheral blood samples from women without breast cancer were involved. Control samples were obtained from blood donor women of the biobanks at the Instituto Nacional de Medicina Genómica (Mexico City, Mexico) and Instituto Nacional de Cardiología Ignacio Chávez (Mexico City, Mexico). All participating women signed the informed consent letter. This study was conducted in accordance with the projects CEI 2015/51 and 24/2016/I, approved in October 2015 and September 2016, respectively, by the Ethics Committee of Instituto Nacional de Medicina Genómica.

### 4.2. Genomic DNA Extraction

Peripheral blood samples were obtained by venipuncture and centrifuged at 1850× *g* to separate leukocytes. Tumor samples were taken during mastectomy and stored at −80 °C until further use. The cellularity of the breast tumors was evaluated, and samples with >80% tumor cells were included. Total DNA from PB and tumor tissue samples was extracted using the Maxi Kit (Qiagen, Hilden, Germany) and AllPrep DNA/RNA MiniKit (Qiagen, Hilden, Germany), respectively, according to the manufacturer’s specifications. Genomic DNA concentration and purity were assessed by spectrophotometry (NanoDrop One, Thermo Fisher Scientific, Waltham, MA, USA).

### 4.3. Direct Mitochondrial DNA Sequencing

The mtDNA was enriched by long PCR using two overlapping primer pairs to avoid contamination with NUMTS, as we reported previously [18]. Paired-end genomic libraries of 150 bp length were generated using the Nextera DNA Flex Library Prep Kit (Illumina, San Diego, CA, USA), and sequencing was performed on the MiSeq platform (Illumina, San Diego, CA, USA). A minimum depth coverage of 300x per base was used.

### 4.4. Mitochondrial DNA Content Quantification

The determination of the mtDNA copy number was realized by quantitative PCR in real time through a standard curve. Specific primers for the mitochondrial *MT-ND1* gene and the nuclear *HBB* gene reference were used. The relative mtDNA copy number was calculated with the 2^−∆CT^ method.

### 4.5. Bioinformatic Analysis

Reads with a depth coverage equal to or greater than 300x per base were analyzed. The quality control of the reads was evaluated with the FastQC algorithm (Braham Bioinformatics) [81]. Reads with a Phred score greater than 25 were considered for alignment with the mitochondrial genome reference (NC_012920.1) using the Burrows–Wheeler–Aligner (BWA) algorithm [82]. The reads were indexed, the duplicates were marked, and the bases were recalibrated with the Genome Analysis Tool Kit (GATK, Broad Institute, MA, USA) [83]. The mitochondrial ancestry was assigned by haplogroups determination using the sequence from blood samples with the mtDNA server Haplocheck algorithms [84,85]. Variant calling was performed with Strelka2 (Illumina Inc.) [86], and those variants flagged as PASS were filtered. To determine the heteroplasmy of the variants, the MAF was calculated based on the depth of sequencing. The variants were defined as heteroplasmic if MAF < 95%, or homoplasmic if MAF > 95% [25,68]. The variants identified in PB of the patients were compared with those detected in control women. To predict the functional effect of the variants, the Variant Effect Predictor (VEP) algorithms were used with default parameters [87]. All variants were searched in the MITOMAP [47] database to find out previously reported or associated with breast cancer or other types of neoplasia.

### 4.6. Statistical Analysis

The total number of variants distributed along mtDNA was counted, and their frequency was estimated considering all the individuals of each group. The number of variants per gene and the frequencies between cases and controls were compared using Chi-square and Fisher’s exact tests. Mutational frequencies, mutational burden, and mtDNA content were correlated with clinical variables (molecular subtype, clinical stage, tumor grade, and survival) of patients with breast cancer. The association of the variants and their risk for breast cancer developing was estimated using the Odds Ratio (OR) with 95% confidence intervals. Overall survival was assessed using Kaplan–Meier curves adjusted to a Cox Regression model. *p*-values < 0.05 were considered statistically significant and were adjusted (*p*-adj) by the Bonferroni method when required. All graphs and statistical tests were performed using the R programming language (R version 4.0.2) [88].

## 5. Conclusions

This research describes the mtDNA germline variants landscape of Mexican patients with breast cancer and healthy women. Our data show that mtDNA alterations in the PB of women with breast tumors differ significantly from healthy women. Although the biological mechanism of these alterations, as well as their relevance in the oncogenesis process, must be investigated in more detail, detection of specific variants and determination of mitochondrial haplogroups could be a tool with high potential for predicting the risk of breast cancer and establishing the prognosis. Longitudinal studies in larger and independent case-control cohorts are required to decipher the potential clinical application of our findings and whether they are meaningful for early diagnosis of breast cancer.

## Figures and Tables

**Table 1:** Clinical features of patients with breast cancer.

Clinical Variable		Frequency *n* (%)
Histological diagnosis	Invasive Ductal Carcinoma	76 (82.6)
	Ductal Carcinoma In Situ	1 (1.51)
	Invasive Lobular Carcinoma	11 (11.9)
	Mixed Carcinoma	3 (3.3)
	NA	1 (1.1)
Clinical Stage	I	8 (8.7)
	IIA	43 (46.7)
	IIB	24 (26.1)
	IIIA	7 (7.6)
	IIIB	5 (5.4)
	IIIC	3 (3.3)
	NA	2 (2.2)
IHC classification	Luminal A	57 (61.9)
	Luminal B	22 (24.0)
	HER2	5 (5.4)
	Triple Negative	6 (6.5)
	NA	2 (2.2)
Metastasis	Positive	11 (11.9)
	Negative	65 (70.7)
	NA	16 (17.4)
Death	Positive	9 (9.8)
	Negative	67 (72.8)
	NA	16 (17.4)

NA: Data not available; IHC: immunohistochemical.

**Table 2:** Number of mitochondrial DNA variants in peripheral blood of patients with breast cancer and controls.

	Controls (N = 75) *n* (%)	Cases (N = 92) *n* (%)	*p*-Value
Total variants	401	866	-
Mutational rate (mut/kb)	24.2	52.3	-
SNVs	378 (94.3)	831(95.9)	0.23 ^a^
Insertions	11 (2.7)	16 (1.9)	0.41 ^a^
Deletions	12 (3.0)	19 (2.2)	0.51 ^a^
Variants per individual ($\bar{x}$ ± SE)	33.6 ± 5.9	46.1 ± 16.8	6.225 × 10^−10^ *^b^
Range of variants per individual	21–49	27–109	-

SNV: single nucleotide variant; $\bar{x}$: mean; SE: standard deviation; ^a^ Chi-square test; ^b^ Wilcoxon test; * Statistically significant values.

**Table 3:** Number of variants per gene/region in peripheral blood mtDNA from patients with breast cancer and healthy women.

	Controls	Cases	
Gen/Region	Total Variants ^a^ N = 402 (100%)	Total Variants ^a^ N = 863 (100%)	*p*-Value ^c^
D-Loop	119 (29.6)	164 (19.0)	2.7 × 10^−5^ *
*MT-ND5*	25 (6.2)	94 (10.9)	0.011 *
tRNAs	13 (3.2)	57 (6.6)	0.022 *
rRNAs	32 (8.0)	103 (11.9)	0.045 *
*MT-CO1*	32 (8.0)	54 (6.3)	0.179
*MT-ND6*	6 (1.5)	24 (2.8)	0.234
*MT-CO2*	14 (3.5)	22 (2.5)	0.443
*MT-ND4*	26 (6.5)	66 (7.6)	0.542
*MT-ND4L*	4 (1.0)	13 (1.5)	0.643
*MT-ATP6* ^b^	26 (6.5)	49 (5.7)	0.651
*MT-CO3*	17 (4.2)	32 (3.7)	0.756
*MT-ATP8* ^b^	8 (2.0)	14 (1.6)	0.803
*MT-ND2*	20 (5.0)	46 (5.3)	0.915
*MT-CYB*	28 (7.0)	58 (6.7)	0.946
*MT-ND1*	23 (5.7)	49 (5.7)	1
*MT-ND3*	9 (2.2)	18 (2.1)	1

^a^ Total number of mtDNA variants does not consider intergenic regions (eight and three variants in cases and controls, respectively); ^b^ variants on *MT-ATP6* and *MT-ATP8* genes may overlap (eight and four variants in cases and controls, respectively, were in both genes); ^c^ Chi-square test; * statistically significant values.

**Table 4:** Total heteroplasmic/homoplasmic mitochondrial DNA germline variants in patients with breast cancer and healthy women.

Group	Heteroplasmy		Homoplasmy	
	Total Vars *n* (%)	$\bar{x}$_MAF_ (Range)	Total Vars *n* (%)	$\bar{x}$_MAF_ (Range)
Controls	85 (21.2)	63.3% (22.2–95.0%)	316 (78.8)	99.8% (95–100%)
Cases	576 (66.5)	10.8% (0.02–94.8%)	290 (33.5)	99.7% (95.1–100%)
*p*-Value ^a^	<2.2 × 10^−16^ *		<2.2 × 10^−16^ *	

Total vars: total of mtDNA germline variants; $\bar{x}$_MAF_: Mean of Mutant Allele Fraction; ^a^ Chi-square test; * statistically significant values.

**Table 5:** Peripheral blood mitochondrial DNA mutational burden association with risk of breast cancer.

Mutational Burden	Controls (N = 75)	Cases (N = 92)	OR [CI]	*p*-Value ^a^
	*n* (%)	*n* (%)		
High (>34 variants)	35 (46.67)	71 (77.17)	3.83 [1.89–7.95]	5.3 × 10^−5^ *
Low (≤34 variants)	40 (53.33)	21 (22.83)		

OR: odds ratio; CI: confidence interval at 95%; ^a^ Chi-square test; * statistically significant values.

**Table 6:** Mitochondrial DNA germline variants associated with the risk of breast cancer.

Variant	Gene/Region	Functional Effect	Controls (N = 75) *n* (%)	Cases (N = 92) *n* (%)	OR [CI]	*p*-Value ^a^	*p*-adj ^b^
A263G	D-Loop	Regulatory: OHR	38 (50.7)	16 (17.4)	0.2 [0.1–0.4]	5.8 × 10^−6^	1.5 × 10^−4^
A16183C	D-Loop	NC	2 (2.6)	21 (22.8)	10.7 [2.5–97.3]	9.3 × 10^−5^	2.3 × 10^−4^
C14766T	*MT-CYB*	Missense: T/I	62 (82.6)	91 (98.9)	18.8 [2.7–816.3]	1.4 × 10^−4^	3.5 × 10^−3^
C7028T	*MT-CO1*	Synonymous: A	63 (84.0)	91 (98.9)	17.1 [2.4–746.2]	6 × 10^−4^	0.02
A235G	D-Loop	Regulatory: OHR, mtTF1 BSX, CSB1	39 (52)	24 (26.1)	0.32 [0.2–0.6]	7.3 × 10^−4^	0.02
G4820A	*MT-ND2*	Synonymous: E	9 (12)	28 (30.4)	3.2 [1.3–8.3]	4.8 × 10^−3^	0.12
G11719A	*MT-ND4*	Synonymous: G	65 (86.6)	90 (97.8)	6.9 [1.4–66.5]	6.5 × 10^−3^	0.16
T16519C	D-Loop	NC	25 (33.3)	50 (54.3)	2.4 [1.2–4.7]	7.9 × 10^−3^	0.16
T16189C	D-Loop	NC	6 (8)	21 (22.8)	3.4 [1.2–10.8]	0.01	0.27
C6473T	*MT-CO1*	Synonymous: I	9 (12)	26 (28.3)	2.9 [1.2–7.5]	0.01	0.32
CT16188C	D-Loop	NC	1 (1.3)	11 (11.9)	9.9 [1.4–437.4]	0.01	0.32
G11914A	*MT-ND4*	Synonymous: T	9 (12.0)	25 (27.2)	2.7 [1.1–7.1]	0.01	0.40
T4977C	*MT-ND2*	Synonymous: L	9 (12.0)	24 (26.1)	2.6 [1.1–6.8]	0.03	0.77
A10398G	*MT-ND3*	Missense: T/A	13 (17.3)	30 (32.6)	2.3 [1.1–4.8]	0.03	0.81
T16325C	D-Loop	NC	13 (17.3)	30 (32.6)	2.3 [1.1–5.3]	0.03	0.81
C10400T	*MT-ND3*	Synonymous: T	11 (14.7)	26 (28.3)	2.3 [1.0–5.6]	0.04	1
T9950C	*MT-CO3*	Synonymous: V	11 (14.7)	26 (28.3)	2.3 [1.0–5.6]	0.04	1
A8701G	*MT-ATP6*	Missense: T/A	17 (22.7)	35 (38.0)	2.1 [1.0–4.4]	0.04	1
C15535T	*MT-CYB*	Synonymous: N	12 (16.0)	27 (29.3)	2.2 [1.0–5.1]	0.04	1
T10873C	*MT-ND4*	Synonymous: P	9 (12.0)	23 (25.0)	2.4 [1.0–6.4]	0.04	1
A302AC	D-Loop	Regulatory: OHR, mtTF1 BSY, CSB2	3 (4.0)	12 (13.0)	3.6 [1.0–20.5]	0.05	1

OR: Odds Ratio; CI: Confidence Intervals at 95%; ^a^ Fisher’s exact test; ^b^ *p*-adj: *p*-value adjusted by Bonferroni method; OHR: H-strand origin replication; mtTF1: Mitochondrial Transcription Factor 1 binding site; CSB: Conserved Sequence Blocks; NC: Non-coding; BSX: Binding sites X; BSY: Binding sites Y.

## Data Availability

The datasets generated and/or analyzed during the current study are not publicly available due to being subject to protection by the Instituto Nacional de Medicina Genómica, but are available from the corresponding author on reasonable request.

## Supplementary Materials

The following supporting information can be downloaded at: https://www.mdpi.com/article/10.3390/ijms26178456/s1.

## Abbreviations

The following abbreviations are used in this manuscript:

BWA	Burrows–Wheeler Aligner
CSB	Conserved sequence blocks
ER	Estrogen receptor
GATK	Genome Analysis Tool Kit
HBB	Hemoglobin Subunit Beta
H2	HER2 positive breast cancer subtype
HER2	Human epidermal growth factor receptor 2
HR	Hazzard ratio
IHC	Immunohistochemistry
LA	Luminal A breast cancer subtype
LB	Luminal B breast cancer subtype
MAF	Mutant allele fraction
MT-ATP6	Mitochondrial ATP Synthase Membrane Subunit 6
MT-ATP8	Mitochondrial ATP Synthase Membrane Subunit 8
MT-CO (1-3)	Mitochondrial Cytochrome C Oxidase Subunit (1-3)
mtCN	Mitochondrial DNA copy number
MT-CYB	Mitochondrial Cytochrome B
mtDNA	Mitochondrial DNA
MT-ND (1-6)	Mitochondrial NADH Dehydrogenase Subunits (1-6)
MT-ND4L	Mitochondrial NADH Dehydrogenase Subunits 4L
mtTF1	Mitochondrial transcription factor 1
NUMTS	Nuclear mitochondrial segments
OHR	H-strand replication origin region
OR	Odds ratio
OXPHOS	Oxidative phosphorylation
PB	Peripheral blood
PCR	Polymerase Chain Reaction
PR	Progesterone receptor
ROS	Reactive oxygen species
rRNA	Ribosomal RNA
SNV	Single-nucleotide variant
TN	Triple negative
tRNA	Transfer RNA
VEP	Variant Effect Predictor
