# Supplementary material for: Mitogenomic Alterations in Breast Cancer: Identification of Potential Biomarkers of Risk and Prognosis
Source: Int J Mol Sci. 2025 Aug 30;26(17):8456. doi: 10.3390/ijms26178456 (PMC12428619; doi:10.3390/ijms26178456)
Supplement: Supplementary file 1 [file ijms-26-08456-s001.zip › ijms-3774697-supplementary.pdf]

Supplementary Information

Table S1. Number of mitochondrial DNA variants in peripheral blood and tumor of patients with breast cancer.

	Blood	Tumor	P-value
Total variants	866	869	-
Mutational rate (mut/kb)	52.3	52.4	-
SNVs	831	831	0.82
Insertions	16	19	0.74
Deletions	19	19	0.99
Variants per individual (x̄±SE)	46.1 ±16.8	42.7 ±11.4	0.39
Range of variants per individual	27 - 109	24 - 90	-

SNV: single nucleotide variant; x̄: mean; SE: standard deviation

Table S2. Mitochondrial DNA copy number in peripheral blood and tumor of patients with breast cancer.

Tissue	n	mtCN (x̄ ±SE)	Min	Max	P-value
Blood	37	30.36 ±24.04	5.01	110.85	0.086
Tumor	37	38.96 ±28.77	3.38	137.29	

mtCN: mtDNA copy number; x̄: mean mtDNA CN; SE: standard deviation; Min: minimum; Max: maximum

Table S3. Mitochondrial DNA copy number in peripheral blood of patients with breast cancer and control women.

Group	n	mtCN (x̄±SE)	Min	Max	P-value
Controls	65	18.5 ±11.7	3.3	49.8	0.027
Cases	70	25.7 ±19.6	5.0	110.8	

mtCN: mtDNA copy number; x̄: mean mtDNA CN; SE: standard deviation; Min: minimum; Max: maximum

Table S41. Peripheral blood mtDNA content association with the risk of breast cancer development.

	Controls N=65	Cases N= 70	OR [IC]	P-value
**mtDNA content**	**n (%)**	**n (%)**		
High (> 14 copies)	33 (50.76)	46 (65.71)	1.8 [0.9– 3.9]	0.08
Low (≤ 14 copies)	32 (49.23)	24 (34.28)		

OR: odds ratio; CI: Confidence Intervals

Table S5. Haplogroups distribution in patients with breast cancer and control women.

Origin	Haplogroup	Controls n(%)	Cases n(%)	P-value (case-control)
America	A	44 (58.7)	41 (44.6)	0.124
	B	9 (12)	21 (22.8)	0.074
	C	11 (14.7)	11 (12)	0.607
	D	6 (8)	11 (12)	0.403
Europe	H	0 (0)	2 (2.2)	0.359
	J	0 (0)	1 (1)	0.581
	R	1 (1.3)	0 (0)	0.422
	U	2 (2.7)	0 (0)	0.237
	T	1 (1.3)	0 (0)	0.423
Africa	L	1 (1.3)	5 (5.4)	0.187
	Total	75 (100)	92 (100)	

Table S6. Correlation of mitochondrial DNA alterations with clinical variables of patients with breast cancer.

Variable	Blood Variants<br>Median (range)	Tumor Variants<br>Median (range)	Somatic Mutations<br>Median (range)	Blood mtCN<br>Median (range)	Tumor mtCN<br>Median (range)
**Age Dx**					
Early (<51 years old)	39.5 (28-109)	37.5 (29-90)	1.0 (0-17)	20.2 (8.1-110.9)	27.9 (3.4-101.9)
Late (>51 years old)	41.5 (27-109)	41 (24-79)	1.5 (0-11)	19.8 (5.0-71.8)	25.6 (14.7-137.3)
p-value	0.36	0.23	0.71	0.6	0.84
**Histological Dx**					
IDC	41 (27-109)	39 (29-90)	1.5 (0-17)	21.5 (5.0-110.9)	24.0 (3.4-137.3)
ILC	42 (32-73)	44 (24-54)	1.0 (0-3)	18.1 (7.1-54.7)	25.5 (14.7-108.3)
p-value	0.89	0.6	0.66	0.78	0.96
**Tumor grade**					
I	43.0 (31-56)	36.5 (31.0-41.0)	1.0 (0-4.0)	15.9 (8.2-41.5)	46.1 (NA)
II	40.0 (27.0-109.0)	39.5 (29.0-90.0)	1.5 (0-17.0)	29.0 (5.0-110.9)	25.5 (3.4-137.3)
III	42.5 (28.0-92.0)	38.0 (32.0-69.0)	2.0 (0-5.0)	21.5 (5.5-72.0)	21.2 (15.7-66.5)
p-value	0.8	0.42	0.58	0.3	0.39
**Clinical Stage**					
I	38.5 (31.0-61.0)	37.0 (24.0-46.0)	1.0 (0-2.0)	40.7 (35.8-110.9)	25.6 (20.7-30.5)
II	42.0 (27.0-109.0)	40.0 (29.0-90.0)	1.0 (0-17.0)	37.7 (8.1-74.6)	25.5 (13.7-66.5)
III	38.0 (32.0-52.0)	37.0 (32.0-52.0)	2.0 (0-11.0)	25.8 (5.5-48.1)	21.6 (3.4-41.7)
p-value	0.17	0.17	0.6	0.17	0.49
**Molecular Subtype**					
Luminal A	42.0 (30.0-109.0)	41.0 (29.0-90.0)	1.0 (0-11.0)	16.7 (5.5-74.6)	24.0 (3.4-137.3)
Luminal B	42.0 (27.0-88.0)	39.0 (29.0-71.0)	1.5 (0-17.0)	29.1 (9.0-110.9)	34.3 (20.8-101.9)
HER2	34.0 (28.0-38.8)	35.0 (31.0-43.0)	3.0 (0-5.0)	38.6 (NA)	NA (NA)
Triple Negative	38.0 (32.0-49.0)	34.5 (24.0-69.0)	1.5 (0-9.0)	26.0 (8.1-41.6)	17.4 (15.7-25.1)
p-value	0.067	0.19	0.81	0.35	0.17
**Death**					
Positive	36.0 (27.0-47.0)	35.0 (24.0-43.0)	2.0 (0-5.0)	24.9 (9.0-40.9)	24.6 (23.6-25.5)
Negative	43.0 (29.0-109.0)	41.0 (31.0-90.0)	1.0 (0-17.0)	34.6 (5.5-74.6)	24.0 (3.4-66.5)
p-value	0.04*	0.0025*	0.75	0.64	0.94
**Metastasis**					
Positive	39.0 (27.0-64.0)	37.0 (29.0-47.0)	1.0 (0-5.0)	33.1 (9.0-72.0)	23.6 (21.2-25.5)
Negative	42.0 (29.0-109.0)	41.0 (24.090.0)	2.0 (0-17.0)	34.6 (5.5-74.6)	27.0 (3.4-66.5)
p-value	0.41	0.1	0.32	0.84	0.68

Dx: Diagnosis; mtCN: mtDNA copy number; IDC: Invasive Ductal Carcinoma; ILC: Invasive Lobular Carcinoma; * Statistically significant values; NA: Not Available.

Table S7. Status of the mitochondrial alterations in patients with breast cancer.

Alteration	Site/Type	Status	n (%)
Mutational burden	Blood	High (≥ 41 variants)	41 (53.95)
		Low (< 41 variants)	35 (46.05)
	Tumor	High (≥ 39 variants)	43 (56.58)
		Low (< 39 variants)	33 (43.42)
	Somatic mutations	High (≥ 1 mutation)	56 (73.68)
		Low (< 39 mutation)	20 (26.32)
Mitochondrial content	Blood	Elevated (≥ 36 copies)	10 (43.48)
		Decreased (< 36 copies)	13 (56.52)
	Tumor	Elevated (≥ 24 copies)	9 (52.94)
		Decreased (< 24 copies)	8 (47.06)

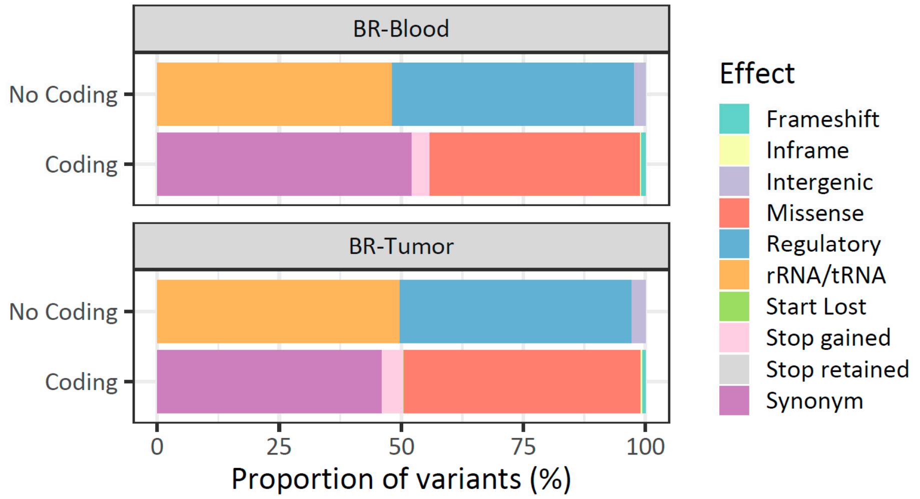

Figure S1. Functional effect of mtDNA variants in patients with breast cancer. Distribution of mtDNA variants in peripheral blood (BR-blood) and tumor (BR-Tumor) of patients with breast cancer is represented. Variants are stratified by their location (coding and non-coding region) and in silico potential functional effect.

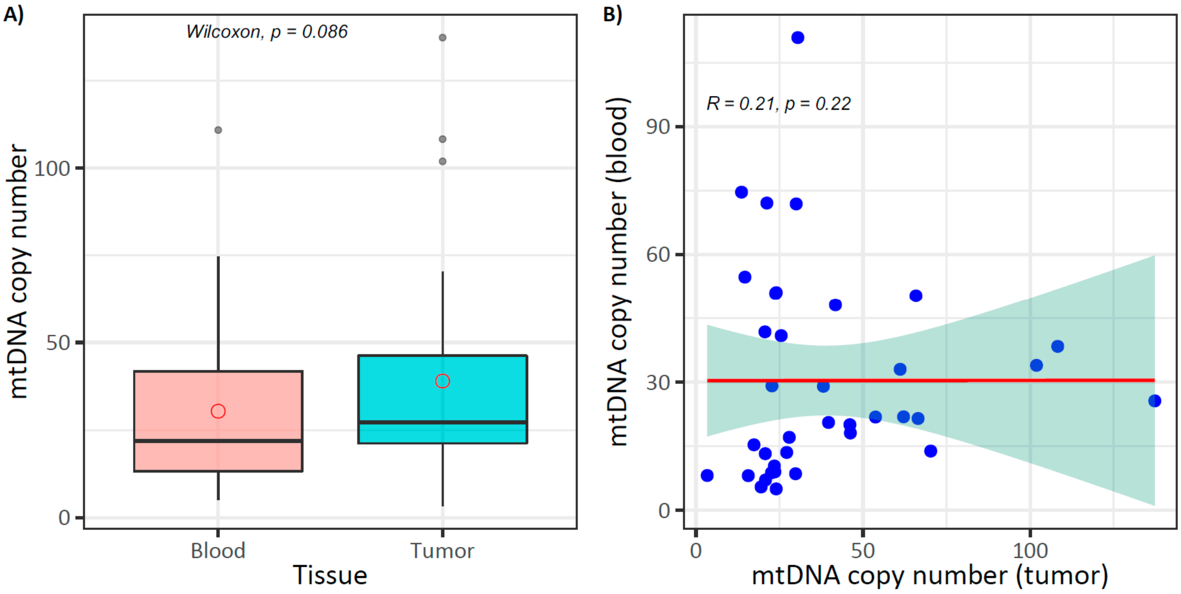

Figure S2. mtDNA copy number in patients with breast cancer. A) Distribution of mtDNA copy number (mtCN) in peripheral blood and tumors of patients with breast cancer. Red circles represent mean mtCN in each tissue. B) Correlation between mtCN in blood and tumor (Spearman correlation, R= 0.21, p= 0.22).

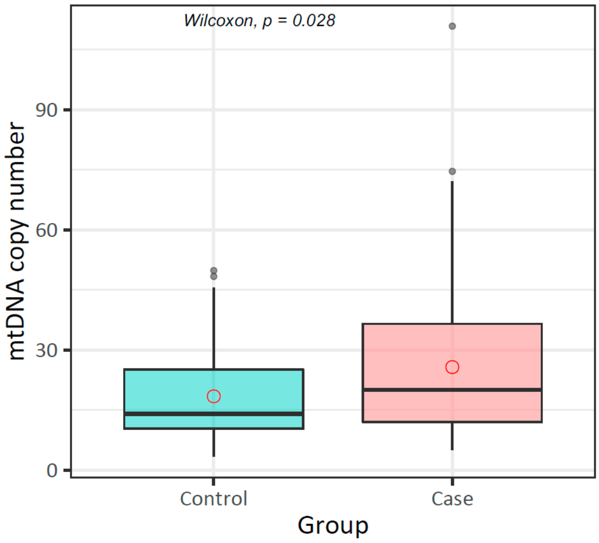

Figure S3. mtDNA copy number in patients with breast cancer and control women. Distribution of mtDNA copy number (mtCN) in peripheral blood of cases and controls is shown. Red circles represent mean mtCN in each group.
